# The business of care: the moral labour of care workers

**DOI:** 10.1111/1467-9566.12184

**Published:** 2015-01-20

**Authors:** Eleanor K. Johnson

**Affiliations:** ^1^ School of Social SciencesCardiff UniversityUK

**Keywords:** residential care, emotional labour, care workers, morality

## Abstract

Drawing on a case study conducted in a private residential care home, this article examines the emotional labour of care workers in relation to the moral construction of care and the practical experiences of work. An examination of the company's discursive attempts to construct, manage and demarcate its employees’ emotional labour was carried out alongside an exploration of the carers’ own interpretations of, and enrolment in, the care‐giving role. The potential economic and emotional consequences of these occurrences were a key focus of the inquiry. The study found that carers, encouraged by the company, naturalised their emotional labour, and that this had contradictory consequences. On the one hand it justified the economic devaluation of the carer's work and left her vulnerable to emotional over‐involvement and client aggression. On the other, it allowed the worker to defend the moral interests of those within her care and to see when those interests were in conflict with the economic motivations of her employer.

Since the publication of Hochschild's work on emotional labour (1979, 1983), there has been an abundance of research examining the emotional aspects of nursing (Gray 2009, Huynh *et al*. 2008, Smith 1992, 2012, Theodosius 2008), midwifery (Hunter 2001, 2004) and care in nursing homes (Lopez 2006). In this body of literature, nurses’ management of emotion has often been characterised as a tacit and uncodified skill that is of potential therapeutic value to patients and nurses (Foster and Hawkins 2005, Gray 2009, Hunter 2001, Phillips 1996), but which typically goes unrecognised and undervalued in healthcare settings (Hunter and Smith 2007, McCreight 2005, Smith and Gray 2000). The invisible character of emotional labour also makes it particularly vulnerable to displacement by new technology (Foster and Hawkins 2005, Rankin 2009), the rationalisation of work (Bone 2002) and a process of professionalisation that has progressively medicalised nursing and, in turn, transferred non‐technical, emotional tasks to those lower down the workplace hierarchy (Apesoa‐Varano 2007, Herdman 2004, Smith 2012). It is in response to these processes of professionalisation, medicalisation and technicisation that researchers have called for an increased explication and codification of the emotional labour entailed in nursing so that it might be reconceptualised and valued as a skill (Gray 2009). Here, the clinical setting in which the nurse carries out her labour; with its high level of professional accountability, rigid structural and regulatory control and increasing technological and medical specialisation, is presented as antithetical to the caring disposition traditionally expected of nurses.

The recognition of the importance of emotional labour in the workplace is not unproblematic, however, for as Hochschild originally observed, it can lead both to feelings of emotional fraudulence and, at the other extreme, the mismeasurement of emotional labour as a natural disposition with its own intrinsic rewards. To examine this further, a case study was undertaken in a private residential home for the elderly; a relatively non‐technological, non‐medically mediated and non‐professionalised space, where one would expect emotional labour to be a central component of the care worker's role. The aim was to explore the extent to which emotions were deliberately put to work in this environment and to consider whether the use of emotion was unambiguously positive for the care workers. The study did indeed find that, unlike nurses in clinical settings, the care workers were actively encouraged by the employer to manage their emotions and that the consequences of this were complex and contradictory.

## Review of the literature

### Defining emotional labour

Hochschild's (1983) pioneering study of flight attendants reflects on the manner in which an individual can actively cultivate and manage emotion, not simply for the purposes of emotional display but also with the aim of bringing one's genuine emotions into line with a society's ‘feeling rules’ – its moral script towards feeling (1983: 56). Here, Hochschild outlines two ways in which we may act: surface acting and deep acting. The former refers to how we outwardly appear to others. In the latter, our display is a product of our work on feeling; it reflects a real feeling that has been self‐induced (1983: 35).

Hochschild's research led her to express concern about the emotional costs for workers when these, formerly private, acts of emotion management come to be bought and sold as a form of labour (emotional labour) and, in turn, instrumentalised and standardised by company‐prescribed feeling rules and rules of display (1983: 55, 186). Hochschild's focus was on the manner in which the commercialisation of emotional exchanges has subordinated a private emotional system to commercial logic and, in doing so, has transformed it, eliminating the worker's right to command what is given in social interaction and to whom (1983: 119, 186). For Hochschild, when a profit motive is placed beneath these aspects of emotional life they are transmuted and it becomes necessary for the worker to relinquish control over how her work is to be carried out, leaving her role deskilled and devalued (Hochschild 1983: 119).

Hochschild's recognition that individuals might be expected to manage their emotional performances in line with an employer's expectations – and her subsequent coining of the term emotional labour – allowed for a proliferation of research on emotion in the workplace. Hochschild's concept of emotion management is particularly amenable to research on the healthcare professions, not least because producing ‘the sense of being cared for in a convivial and safe place’ (Hochschild 1983: 7) has been considered a fundamental element of patient care (Bolton 2000, 2001, Bone 2002, Hunter 2001, 2004, James 1992, Smith 2012, Theodosius 2008). A common assumption in the healthcare literature is that the quality of nursing care is, in part, achieved by means of nurses’ emotional commitment to those in their care (Huynh, Alderson, and Thompson 2008). Likewise, both patients’ complaints and wider public concern over poor quality care have been framed as a response to a lack of emotional sensitivity on the part of healthcare professionals (Fletcher 2000, Strauss *et al*. 1982).

Researchers have commented upon the invisible nature of emotion work in nursing which leaves such work poorly acknowledged and, in turn, undervalued both in medical organisations (Hunter and Smith 2007) and by nurses themselves (McCreight 2005). Emotions have been presented as being in contention with the dominant technical‐rational paradigm in healthcare settings and thus as in danger of being pushed aside in favour of the more physical and technical aspects of care (Hunter 2001). By conceptualising emotion as a freely given element of nursing work that is regulated by the emotional labourer herself, those researching the nursing professions have tended to conceptualise emotional labour as a positive thing which must be protected and left untouched (McClure and Murphy 2007). The result has been an idealising of the emotional elements of caregiving over and above the technical and medical aspects of nursing work, and a neglect of Hochschild's original distinction between emotion work and emotional labour and, with it, her critical insights into the commodification of the emotions.

### Moral reasoning

In order to assess the emotional labour of care workers in a more critical fashion, the research draws upon Ralph Fevre's work (2000, 2003, Fevre *et al*. 2003), which places Hochschild's discussion of the commodification of emotion within the broader context of demoralisation, where economic rationality has come to undermine genuine morality.

For Fevre (2000: 142), society has four distinct sense‐making fields at its disposal, each of which uses a discrete combination of ontology and epistemology. In western societies the forms of sense‐making arising in each of the four categories with which we are most familiar are science, common sense, religion and sentiment (Fevre 2000: 141). The field of sense‐making drawn upon in each instance rests upon the manner in which the knowledge and belief systems surrounding it have been socially constructed (Fevre, Chaney, and Stephens 2003: 4). Both social institutions and individual actors thus have a role in the formation of sense, and each is prone to making a mistake in identifying which sense‐making field is appropriate in given situations – to making what Fevre terms a ‘category mistake’ (2000: 154).

Fevre's account of demoralisation predominantly focuses on two forms of sense‐making: sentiment and common sense. He defines sentiment as a form of interpreting reality that makes a consideration of human feelings (a consideration that rests not on knowledge but on belief) central to the formation of the behavioural guidelines that are usually described as morals (Fevre, Chaney, and Stephens 2003: 3). Conversely, when we draw upon common sense, our reasoning promotes sensations over feelings; our actions are guided by the knowledge yielded by human experience, on the ‘proof’ provided to us by our senses, rather than on belief. ‘We do not have to believe in our senses to sense, but we do have to believe in our feelings to feel’ (Fevre 2003: 73). As knowledge has expanded at the cost of belief, Fevre argues, common sense has spread into areas of life where sentiment is critical to the sustenance of moral reasoning, feelings being the chief casualty of the process of demoralisation. Fevre suggests that demoralisation also results from sentiment being demanded in the service of (common sense) economic reasoning, as when emotional labourers mistakenly moralise transactions that are fundamentally regulated by common sense calculations and instrumental self‐interest.

Fevre uses his concept of sentiment to suggest that Hochschild's (1983: 18) discussion of emotional interactions as gift exchanges guided by feeling rules points towards standards which arise from a morality. For Fevre (2003: 76–7), what is missing from Hochschild's account is the clarification that the corporate feeling rules she examined were being used to substitute for moral rules. It is in this sense that Hochschild's (1983: 186) suggestion that a profit motive has been slipped in under acts of emotion management can be understood as a category mistake which acts to persuade employees that the commercial transaction in which they are engaged is one in which moral rules of behaviour apply – one which must be interpreted in terms of sentiment (2003: 78). Fevre's proposition is that Hochschild's research is essentially a documentation of the creation of an ‘ersatz morality’ in the airline industry, which was able to serve in the place of the genuine morality that had been torn down, in part, by common sense reasoning (2003: 83). The emotional dissonance which Hochschild identified in her flight attendants, whereby workers felt a sense of estrangement between self and feeling, can thus be re‐characterised as moral dissonance – as a symptom of demoralisation (Fevre 2003: 81).

### Research questions

If the emotional labour of the flight attendant is an ersatz morality, then this may well be because her work entails a commercial transaction where the crew's personal services are more a matter of added value than a core element of the business. But what of the case where the client is less a customer and more a person with a need for care? Does sentiment have a more legitimate role to play in care work than in the work of flight attendants? Can care workers avoid the moral dissonances referred to above and enjoy a properly recognised and valued commitment to emotional labour? This research set out to answer these questions.

## Methods

### Research setting

The findings draw upon a case study carried out in a private residential home, located in the south of England, which I am calling Oakwood. Founded in the 1980s, Oakwood forms part of an international, publicly traded company that operates at the high‐end of the social care market. Oakwood was initially chosen because it offered the greatest opportunity to learn, because of my ability to gain access to it as a past employee. Further, unlike residential homes at the lower end of the care home market, the training of new employees and day‐to‐day practices at Oakwood were known to revolve heavily around a managerial discourse of ‘the good worker’. The company's explicit promotion of emotional labour allowed it to be examined as a means to gain further insight into this issue. Methodological choices were thus made with the intention of both refining and supplementing previous theoretical arguments, illustrating if, how and in what manner those concerns identified in the literature were manifest in the case. Access to the site was negotiated with the managing director of the residential home.

### Research design

Data collection was carried out between June and September 2012. The first stage of the research involved the analysis of a corpus of documents that was purposively selected in order, firstly, to examine the external impression which the company intended to give of itself and its workers and, secondly, to examine the manner in which it defined and constructed carers’ emotional labour. The document analysis thus comprised two parts. Firstly, publically available documents such as the company's website, newspaper advertisements and brochures given to prospective clients were examined. Secondly, carers’ job specifications and training manuals, as well as Oakwood's company handbook, which was given to all new employees, were examined.

The resulting findings were used to guide the subsequent stage of research: participant observation. These observations focused on the company's week‐long induction training of new employees, which was carried out by a regional trainer. I adopted the role of a new recruit, though both the regional trainer and the new recruits were made aware that I was carrying out the research. Each new recruit provided informed consent prior to the observations being undertaken and several agreed to be interviewed following the training. The observations were conducted with the intention of examining the feeling rules that the company attached to the care‐giving role, the techniques it used to encourage the carers to carry out their emotional labour and the extent to which this involved a form of ersatz morality.

Following the observations, semi‐structured interviews were conducted with six new recruits who had attended the training, five of whom were women. The principal themes of these interviews were: the participants’ motivations for becoming carers, their first impressions of their employer and their thoughts about Oakwood's service principles, and the role of feelings and the performance of feelings. Interviews were subsequently undertaken with six care workers who had worked at Oakwood for over 12 months, five of whom were women. The key themes of these interviews were whether their initial feelings about their work had changed, how they defined doing a good job, their experiences of working for the company, their interpretations of its service principles, and the challenges and dilemmas that could arise at work.

### Data analysis

Each interview was transcribed, alongside any related field notes, and a summary of each carer's account was written down in order to avoid the decontextualisation and fragmentation of the respondents’ narratives. The transcripts, observation notes and key extracts of text from the document analyses were uploaded to NVivo and coded thematically. An adaptive approach was employed. Here, data was analysed both deductively, in terms of considering if those concerns previously identified in the literature arose within the data, and inductively, whereby interpretations of the data were used to rework and augment the existing theoretical framework (Layder 1998). Each code denoted a key theme, which will now be discussed.

## Findings

### The naturalisation of emotional labour

In a handbook given to new employees, Oakwood stressed that those whom it chose to employ were ‘truly unique’ in that they held the belief that ‘real happiness and joy come from serving others’; they had a ‘natural serving heart’ and were ‘born to care’. Likewise, several respondents described this altruistic disposition as an integral part of their nature, not just a marketable skill but something which they were used to acting upon in private settings. For example, Irena, a new recruit said ‘if you ask my family “what are my qualities?” then they say I'm very caring and very loving and nurturing. I think I have it naturally within me’. Equally, Miranda noted:

I believe that I should help those who are weaker than me. I think that unless you believe in those things it will be difficult to acquire them whilst you're working … It's like something that shines. It's who you are.

Both Ehrenreich and Hochschild (2002: 76) and Payne (2009: 349) have suggested that the construction of workers’ care activities as something that requires what are frequently deemed to be ‘natural’ feminine qualities (such as nurturing and empathy), causes the skill content of such work to go unnoticed and poorly rewarded, as the labour that those activities entail is effectively erased. This was clear when Jane, an experienced carer, said ‘I personally feel that what I do is natural, I don't have to try’.

Despite receiving an extremely low income in comparison to other full‐time workers (the weekly earnings of a care worker working 35 hours a week at Oakwood were £234 while the national median was £506 [ONS 2011]) – just one respondent, Rachel, an experienced carer, felt that her wage was unreasonable. Furthermore, when interpreting the appropriateness of their wages, the carers seemed to place added value on the technical and learnt, as opposed to emotional, aspects of their work. Rachel, for example, felt that £7 an hour was a reasonable wage for those ‘doing basic care like talking to people and helping get them up’, as these activities did not constitute ‘doing more than average’ and certainly involved ‘much less than doing meds’, which required training. Likewise, of the 11 respondents who felt that their wage was reasonable, three said that they would expect a higher salary if they held care‐related qualifications and four mentioned that training as a medical technician would lead them to expect higher wages. It appeared that, because the carers had naturalised a key aspect of their role – emotional labour – they undervalued their work and felt that, without emphasising the technical and learnt aspects of their role, they would have weak grounds for expecting higher wages. This replicates the perspective of nurses interviewed by Gray (2009) who viewed the model of the natural, female carer as a barrier to the establishment of nursing as a profession. The consequence, as Smith (2012: 188) observes, is a hierarchy of values between personal care activities and technical skills in nursing, whereby personal care activities are frequently demoted down the occupational ladder. Smith (2012) warns that this hierarchy is illustrative of Hochschild's (2003: 221) contention that the ‘cold modern ideal’ of providing the most practical, efficient and rational means of care will prevail if the emotional aspects of care continue to be undervalued and under‐rewarded.

### Motivations to care: altruism as a resource

At Oakwood, however, rather than portraying emotion as a weakness (Gray 2009) to employees, the company implied that workers who acted upon their ‘natural’, altruistic motivations would find their work rewarding in its own right. For example, the company handbook promises employees that ‘the best benefit of working at Oakwood is the feeling that you are making a difference in so many lives’. Five of the six new recruits described their motivations to become carers in terms of how the role would allow them to express their philanthropic nature and realise their true self. Wendy, for example, who had previously cared for her mother, stated: ‘I need to pass that care I have on to somebody else’ and suggested that care‐work would allow her to do this, providing ‘that double whammy… a career and job satisfaction as well’. Miranda noted ‘I believe that I should help those who are weaker than me… so I would actually prefer to do this work if I can, because I know that it will give me more satisfaction’. Likewise, five of the experienced carers described the ‘fulfilment’ and ‘satisfaction’ that they felt when ‘going home exhausted’ (Julie) or when leaving residents ‘happy, smiling [and] reassured’ (Jane). It was clear that most of the new recruits and experienced carers took a vocational, as opposed to instrumental, attitude towards their employment.

Folbre (2002) and England (2005) have argued that if workers consider the intrinsic properties of their work as an amenity for themselves (in this case, if they find satisfaction in caring for people) then this makes them more prepared to accept a lower wage. The manner in which Oakwood made use of its recruits’ altruistic motivations was particularly apparent when the company discussed employee benefits, which included ‘hugs from the residents’ and ‘“thank‐yous” from their families’. By defining the carers’ work not only as natural but as naturally remunerated in the same moral currency of hugs and thank‐yous (in what Oakwood's CEO unashamedly refers to as a ‘second paycheque’ in a video shown to new recruits) the company further undermined its employees’ capacity to dispute their pay packets by causing them to evaluate their work in terms of sentiment. The experienced carers certainly appraised their day‐to‐day work performance in terms of whether they had been able to gain a sense of personal worth and moral value from their clients or their clients’ families. When asked to describe an instance where she felt that she had done a ‘good job’, Jess said ‘the family were really grateful, they really appreciated it… it made you feel like you're really doing something worthwhile’. Rachel also noted that ‘you get more out of your day if you push yourself to be positive and make that person happy’. Rachel's strategy to being positive at work was to put herself ‘in the family's position … so that you kind of forget about yourself’. Encouraged by the company, carers at Oakwood identified their work as something that they genuinely enjoyed and, in turn, found themselves actively participating in reducing the value and visibility of their labour.

This runs counter to Theodosius’ observation that the quasi‐marketisation and privatisation of health care in the UK has challenged:

The belief that the nurse is caring because she is expressing something that is intrinsic to her fundamental character, freely offering care to her patient in exchange for gratitude and personal satisfaction. (2008: 39)

At Oakwood, this feeling rule, which works on the assumption that ‘emotional labour is representative of [carers’] desire to care and is something that is … freely given’ (Theodosius 2008: 37) was not challenged or undermined but actively encouraged.

### Deep acting: the management of feeling rules

Theodosius’ research on the emotional labour of nurses also proposes that, when the relationship between nurse and patient is commercialised, there is a loss of ‘the significance of feeling rules that mediate issues of intimacy between nurses and patients by imitating personal and private relationships’ (2008: 39). For Theodosius, this causes nurses to adopt a more cynical view of their emotional labour and results in the substitution of deep acting with surface acting. At Oakwood, however, training hand‐outs strongly encouraged recruits to behave towards residents ‘as if’ they were family or close friends, stating that they must ‘develop a genuinely close relationship with the resident’ ensuring that the home would feel like:

A safe, loving place where the resident would be among friends … [and] where residents and staff can curl up on the sofa together to share laughter as well as tears.

For Hochschild (1983: 7) the induction and suppression of feelings required to produce ‘the sense of being cared for in a convivial and safe place’ often draws upon a source of self that we honour as deeply integral to our personality. In the case of Oakwood, the company not only marketed the convivial nature of its employees to potential clients, it depicted the client–carer relationship as entailing a deep, authentic, emotional bond. In consequence, when asked whether their level of commitment had changed since first being employed at Oakwood, all the experienced carers said that their level of commitment had increased and each attributed this to the personal bonds that they had formed with residents. These findings run counter to the suggestion that the commercialisation of health care has led healthcare workers to adopt a more cynical attitude towards their emotional performances (Theodosius 2008: 39), partaking in only surface acting as a means to disguise the mechanistic and un‐individualised nature of hospital care (Herdman 2004, Wigens 1997). In fact, Oakwood's corporate strategy, by establishing feeling rules that encouraged the imitation of familial care‐giving, acted to augment the carer's sincere adoption of the care‐giving role.

When the carers were encouraged to treat the residents as if they were family, the company was, in Hochschild's terms, advocating the specific imaginings that they might draw upon in their interactions with the residents (1983: 49). Hochschild warned that one consequence of such recommendations is a commercial colonisation of imaginative resources, the effect of which may be confusion between self and role (and, in this case, a nagging disquiet about who exactly one's family really are). What was most noticeable here, however, was how the company's emotional overtures acted to persuade its workers to over‐identify with the care‐giving role at the expense of upholding their political and economic entitlements as workers. By encouraging the carer to apply sentiment to her work (by causing her to identify it as natural), Oakwood invited her to make what Fevre (2000: 154) would term a category mistake. The carers’ satisfaction with their wages implies that they had made this category mistake and, in consequence, struggled to give up their care‐giving role in favour of adopting an ‘uncaring’, dispassionate, common sense relationship with their employers that would be a pre‐requisite for expressing their economic entitlements as workers.

### Voluntarism

The experienced carers’ tendency to evaluate their work in moral, as opposed to economic, terms was clear in their accounts of when they had done a good job. Here, by evaluating their work in terms of the moral currency of emotion, the carers had a tendency to give of themselves voluntarily. Pierre, for example, noted that he appraised his work in accordance with the company's feeling rules by thinking to himself ‘this is my parent or my relative’. For Pierre, this meant that doing a good job inevitably entailed ‘doing more than what you're told to do’ or ‘being able to stay after work’. Likewise, Sarah referred to an occasion where ‘I came in, in my own time. I didn't get paid for it… and she [the resident] was really thankful for it and that made me happy’.

These acts align with what Bolton and Boyd (2003: 293) would term ‘philanthropic emotion management’, which refers to workers’ activities that are motivated by a choice to surpass organisational prescriptions, displaying additional emotion to what a company requires in the form of a gift. In their critique of Hochschild, Bolton and Boyd use this term to help demonstrate how emotional displays in the workplace do not emerge from only one kind of emotion management – that governed by a company's profit motive (2003: 296). Bolton's (2000, 2001) own research has outlined instances of such autonomous, ‘authentic’ emotion management in the nursing professions. She presents this gift of emotion work as something that is becoming increasingly difficult to offer in marketised healthcare services (2000). What was clear in the case of my respondents, however, was that, while these acts of emotion management were voluntary, the social feeling rules on which they drew had come under the sway of the company. For example, in the quotes above, though both Sarah and Pierre described instances where they had provided care beyond that specified in their contracts, they evaluated that care in terms of Oakwood's prescribed feeling rules which defined good care as ‘family‐like’ and as intrinsically, as opposed to financially rewarding work.

Like Hochschild, Gorz (1989) argues that there is a fundamental transformation of the meaning of certain activities when they are subjected to commercial logic. For Gorz, a carer must not have a vested interest in people requiring her to care; her job is done well only when it is performed out of a sense of vocation; an unconditional desire to help others (1989: 144). That there is economic demand, and therefore remuneration, for the care that she offers should not be the carer's basic motivation; money simply enables her to fulfil her vocation. There is therefore, in Gorz's view, always an element of care that takes the form of a gift, whereby the receiver of care receives something from the carer which is beyond that which is, or can be, remunerated (1989: 144).

When the company becomes overly prescriptive in defining the carer's work – when it excessively manages the feeling rules to which she must refer – two possible consequences arise. One is that the gift is withdrawn, the quality of care atrophies and interpersonal labour degenerates into ‘soulless conviviality’ (Gorz 1989: 145) much like that characteristic of Hochschild's ‘cold modern solution’ (2003: 221). The other is that the carer continues to give – she continues to care – but in a commercial context that makes that care servile and potentially humiliating. In those activities where the very gift of oneself becomes the subject of the commercial exchange: when, in this case, the company attempts to teach, purchase and manage the act of giving; this gift is necessarily devalued or distorted. There is, Gorz argues:

An inalienable dimension of our existence, the enjoyment of which we cannot sell to anyone without *giving of ourselves* into the bargain, and the sale of which devalues the act of giving without relieving us of the obligation to perform it as a gift. (1989: 149).

Oakwood seemed to understand that it could successfully manage, commercialise and cheapen the provision of care without destroying the workers’ obligation to perform it as a gift.

### Maintaining a smile: surface acting

Also key to Oakwood's managerial philosophy were its service principles, which were displayed ubiquitously in the company's advertising and in training hand‐outs. They were defined as Oakwood's hallmark, which functioned to guide employees in all their work interactions. The company's induction training made several calls for surface and deep acting which drew upon the normative framework established by these principles. One such call for surface acting was the need to maintain a smile. When discussing the principle of ‘joy in service’, Peter, the trainer, said ‘people want to come in and see happy staff … You walk through the door and BOOM, it's Oakwood … I'm an actor’. The need to maintain a smile at work was the topic of much discussion in the interviews with new recruits, who all stated that being positive at work was important.

The new recruits recognised that this kind of performance might not reflect their immediate feelings. For example, Wendy described herself as ‘a firm believer that you hide behind your smile’ and Josie said ‘as soon as you walk in … if you've got problems you put a front on … It's important that you try and do that’. Inadvertently employing Goffman's terminology, Josie referred to smiling at work as a ‘front’ that should be assumed upon entering the workplace, what would be her front‐stage. Although in Goffman's (1959: 28–32) terms her attitude could be categorised as cynical, Josie's willingness to play this role combined egocentric motivations with a deeper and more compelling moral inflection. Though conceding that not being ‘outgoing’ and not ‘putting on a smile’ at work might ‘get her into trouble’, Josie also understood the need for surface acting in terms of her deep emotional beliefs, which she saw as ‘part of being caring’, describing the display of negative emotions to the residents as ‘wrong’.

All the experienced carers also linked being a good carer with adopting an insincere display of positivity – to being an actor, as Peter recommended. For example, Rachel stated ‘you leave your problems at the door … if you come in miserable then you're in the wrong job … that's what we're about’. Jane, when asked what she thought about the need to maintain a smile, said:

I think it's brilliant because I'm a smiley, caring person anyway … if you smile at them, if you're genuinely happy, even if you're not really happy, if you've got a smile for the residents then it lifts their spirits.

Jess, similarly, when asked if it was always possible to be positive at work, said ‘I think it comes naturally to me I mean … you should act like you're always happy … because it makes the residents more happier’.

This diverges from the experiences of Goffman's medical students, who were required to drop their idealistic orientations to their work upon commencing medical school, laying aside their holy aspirations of showing concern for people with diseases (1959: 31). Instead, the carers’ motivations and aspirations were put to use by the company, with carers regarding their adherence to the company's prescriptions for surface and deep acting as the realisation of these motivations. Thus, while acknowledging that being positive at work would entail the maintenance of a front – surface acting – the carers sincerely defined the assumption of this front as a reflection of self; an offshoot of their ready‐made capacity to care. This kind of surface acting is different from the cynical performances of service workers described by Goffman who are ‘sometimes forced to delude their customers because their customers show such a heartfelt demand for it’ (1959: 29). Instead, it is the care workers who have the ‘heartfelt demand’ – their surface performance is in the service of a deeper conviction – a deep feeling rule – about the moral importance of care. The surface acting of the carers thus departed from Hochschild's (1983: 36–7) definition of the phenomena as an affective display which is put on, where the actress is ‘acting as if she had a feeling’. Rather than leaving a separate, cynical self intact, the carers’ surface displays were a product of their sincere commitment to their caring role, which the carers took on as a component of self. Some of the implications of this sincere commitment will now be explored.

### Carer vulnerability

In Hochschild's (1983) analysis of flight attendants, acting in the interest of the client often meant tolerating objectionable behaviour, so the rules of moral conduct were unequally loaded in favour of the customer. Like the client–worker relationship in Hochschild's study, the carer–resident relationship is one‐way: it requires that the carer gives of herself without expecting anything from the resident in return. This one‐way personalisation was apparent in several of the feeling rules that the company prescribed to the carers, including the need to be positive and smile at work.

In the context of the residential home, the dependency of the clients on the carers makes objectionable behaviour of the ‘demanding customer’ type, as was experienced by Hochschild's flight attendants, a less common occurrence. Nonetheless, the carers did report dealing with challenging behaviour on a regular basis. In the induction training at Oakwood, Peter did little to define what client behaviour might be unacceptable, justifying the need for emotional labour not in terms of the residents’ payment for a service but in terms of their impaired mental capacity, physical vulnerability and his assumption that ‘you can't treat an old dog new tricks’. In the training, any discussions about interactions that might upset, anger or harm the carers emphasised how the carers should manage their own emotions rather than the behaviour of the residents.

Although caring for vulnerable people is very distinct from the stewarding of airline clients examined in Hochschild's work, there is a danger that the one‐way nature of the carer–client relationship can be extended or taken advantage of. In the case of Oakwood, the vulnerability of the clients was, quite rightly, considered as a legitimate basis for the carers to manage their own emotional displays. It was when these one‐way rules of moral conduct were extended to those occurrences where the residents verbally or physically mistreated the carers, however, that the latter were placed in a potentially vulnerable position. For example, Jane, an experienced carer, mentioned a client's aggressive behaviour:

She was obviously having a distressing moment for her and she screamed at me and called me a fat bitch. But umm [laughs] … I think you have to try not to let it affect you, I mean they've got memory problems so they probably don't realise what they're doing … as long as you keep smiling! [Laughs].

Likewise, other carers referred to residents who ‘shout at you all the time’, who were ‘quite forceful with you’ (Rachel), and instances where residents had ‘lashed out’ and ‘kicked them’ (Pierre). In accordance with Luck *et al*.'s (2008) study on nurses’ ascription of meanings to their patients’ acts of violence, it was clear that the carers at Oakwood condoned the ill‐treatment that they were subjected to in terms of ‘mitigating factors’ such as the residents’ reduced responsibility. Though the carers admitted that it was sometimes challenging to care for certain residents, the fact that those residents ‘might have dementia’ or ‘suffer from confusion’ meant that the carers’ response was to ‘just get on with it’ and ‘deal with it yourself’ (Rachel), to ‘learn to try and deal with things’ and ‘let things go over your head’ (Jess), or to ‘try and ignore it’ (Pierre). For Luck *et al*. (2008: 1076), while ascribing these meanings to clients’ violent behaviour allows the worker to remain calm, tolerant and sympathetic towards the client, the long‐term consequence is that such events go under‐reported and under‐recorded. This, in turn, makes it harder to challenge one‐sided training protocols and to expose the failure of companies like Oakwood to address the deeper roots of resident misbehaviour, such as deficiencies in care caused by short‐staffing.

### Emotional distress

Just one experienced carer, Jane, said that, despite believing that her emotional obligations to the residents were the same as those that she had towards her family, she was able to ‘go out of the staff door at night and forget about it’. The five other experienced carers described the complex emotional costs of their difficulty to detach themselves from the care‐giving role that they identified as a component of self. These carers not only performed their role as if the residents were close friends or family members; they considered their relationship with certain residents to be wholly personal and authentic. In consequence, they formed deep emotional attachments to the residents. When asked if she felt that she needed to be emotionally involved in her work to do it well, Sarah said:

At the end of the day, if you don't feel emotional you don't care, and then you're not a carer. It's like when residents die and you get some people who are like ‘Oh you'll get used to it’ and I think ‘Well … I'm showing my emotion because I *care’* … you *do* get emotionally attached to everyone.

Several other carers agreed that ‘if you weren't emotionally involved then you wouldn't be very caring’ (Jess), and that ‘you've got to show some kind of emotional attachment to everyone to be able to care properly’ (Rachel); which made Rachel ‘get upset to see them upset’ and resulted in her ‘going home and worrying about the residents’. The carers’ sincere commitment to the care‐giving role – their unreserved application of sentiment – left them at risk of becoming over‐involved in their work. The result was that the carers became emotionally distressed when the residents suffered or died. For example, likening the emotional obligations that she felt towards residents to those that she felt towards her family, Sarah said:

When Pearl died I cried all day non‐stop like it was my granddad. I came home and I cried, I cried before she died, seeing her crying, going home crying. That felt like it was a family member … sometimes I've really, really, really, really, really cried for a resident.

Like Hochschild's flight attendants, identifying so sincerely with their clients left the carers at Oakwood at risk of becoming emotionally exhausted; of being unable to estrange the self from the care‐giving role, which the company constructed as familial (1983: 132). Nonetheless, the carers recognised that it was not possible to retain the same level of emotional involvement with all residents, which perhaps prevented them from entering the state of emotional numbness described by Hochschild (1983: 188).

### Moral advocates: being critical of the company

Of the six experienced carers, those who appeared to most personalise their relationships with the residents (Sarah, Rachel and Jess) were also more likely to express scepticism over the moral integrity of the company. The sincerity with which they undertook their caring role; the heartfelt use of sentiment which they brought to it, was, ironically, what seemed to have led them to adopt a more critical attitude towards the corporate strategy of Oakwood. This criticism was particularly apparent when the experienced carers discussed the company's service principles. For example, Jess said:

It feels like you're on your own in trying to care for the residents really. And you try and express your word to the management but they don't really understand. They say all that stuff about respect and that … but they're just worried about the money and you're trying to care for these residents and you can't give them the right care and it's like … it's battling.

Other examples that carers gave of instances where they felt that Oakwood had gone against its own service principles included encouraging carers ‘to get residents up quickly’ (Sarah); being unable to carry out all a resident's wishes (Jess); and cutting down the amount of time that carers were allowed to spend with residents (Rachel). These problems, which left the carers ‘feeling bad’ (Jess) ‘in a difficult position’ and ‘feel[ing] quite rubbish about what you do’ due to ‘letting down’ residents and their families (Rachel), were attributed to the inadequate staffing levels at Oakwood. The effect of ‘being short staffed’ on the ability of the carers to fulfil the caring role was an issue raised by all the experienced carers. This was briefly touched upon by Peter, the trainer, during the induction: ‘It's a cost balance’, he said. ‘You as care workers would want more staff all the time but it's about doing your job to the best of your ability’.

### Ersatz morality

It appeared that the carers’ unreserved application of sentiment to their work, although being in one sense a category mistake that made them vulnerable to economic exploitation, was also what allowed them to feel a sense of moral righteousness in defending the moral interests of the residents against the commercial interests of their employer. By sincerely identifying their caring role as an aspect of their character that was resistant to social control, the carers felt justified, in the interviews at least, in criticising their employer, albeit by acting as moral advocates for the residents.

This is distinct from the workers discussed by Fevre (2003: 92) who opposed managerial legitimacy by turning ersatz moralities against managers as a tactic to expose or subvert their probable intentions. While the workers’ sceptical attitude towards the company did make use of the ersatz morality fostered by Oakwood, they did so not only as a means of revealing Oakwood's commercial goals, but also in the name of a more authentic morality. The carers’ criticisms of the company called for the replacement of the managers’ common sense reasoning with sentiment – they were effectively asking their employers to take a properly moral stance towards the residents. In this sense, the internalised service principles and related feeling rules had been turned around against the company – in thought if not in deed.

The irony in this situation was that, by making the residents the sole subjects of their moral concern, the carers had effectively suppressed their own moral interests, even refusing to complain about their own ill‐treatment at the hands of difficult residents. Furthermore, as the carers’ altruistic reasoning had caused them to disregard their economic interests, they also stood in danger of inviting the company to address their concerns by shifting the burden of care back to the already overworked carers – that is, by urging them to work harder in order to ensure that their virtuous aspirations of providing good quality care were realised.

## Conclusion

The starting point for this study was the concern that care‐giving in healthcare settings is at risk of being shorn of its emotional elements, which are difficult to cost and measure (Smith 2012: 186–7). The study found that, in accordance with previous research on the emotional labour of healthcare workers (Smith 2012, Theodosius 2008), the company in question undervalued and inadequately rewarded its employees’ emotional labour, but that this did not result in the withdrawal of emotional labour or sentiment: the care workers continued to offer the gift of care (Gorz 1989). Fevre's work (2000, 2003 may offer an explanation as to why emotional labour, in this residential home at least, has endured despite its lack of financial recognition. Here, encouraged by the company's ersatz morality, the carers’ naturalised their emotional labour and continued to act with sentiment. Although this led them to devalue their own interests as employees, it did provide them with a moral language to defend the interests of the residents and recognise when they were at variance with those of the employer. In effect, the ersatz morality of the company had become, for them, a genuine morality – albeit one that made them vulnerable to greater economic exploitation. Here, perhaps, is the unresolvable contradiction of care work in a capitalist society, which is that one cannot care well without the use of sentiment, but one cannot be paid well without the use of common sense.
